# Differences in placental capillary shear stress in fetal growth restriction may affect endothelial cell function and vascular network formation

**DOI:** 10.1038/s41598-019-46151-6

**Published:** 2019-07-08

**Authors:** Win M. Tun, Choon Hwai Yap, Shier Nee Saw, Joanna L. James, Alys R. Clark

**Affiliations:** 1 Auckland Bioengineering Institute, University of Auckland, Auckland, New Zealand; 2 Department of Biomedical Engineering, National University of Singapore, Singapore, Singapore; 3 Department of Obstetrics and Gynaecology, Faculty of Medical and Health Sciences, University of Auckland, Auckland, New Zealand

**Keywords:** Cell biology, Computational biology and bioinformatics, Structural biology

## Abstract

Fetal growth restriction (FGR) affects 5–10% of pregnancies, leading to clinically significant fetal morbidity and mortality. FGR placentae frequently exhibit poor vascular branching, but the mechanisms driving this are poorly understood. We hypothesize that vascular structural malformation at the organ level alters microvascular shear stress, impairing angiogenesis. A computational model of placental vasculature predicted elevated placental micro-vascular shear stress in FGR placentae (0.2 Pa in severe FGR vs 0.05 Pa in normal placentae). Endothelial cells cultured under predicted FGR shear stresses migrated significantly slower and with greater persistence than in shear stresses predicted in normal placentae. These cell behaviors suggest a dominance of vessel elongation over branching. Taken together, these results suggest (1) poor vascular development increases vessel shear stress, (2) increased shear stress induces cell behaviors that impair capillary branching angiogenesis, and (3) impaired branching angiogenesis continues to drive elevated shear stress, jeopardizing further vascular formation. Inadequate vascular branching early in gestation could kick off this cyclic loop and continue to negatively impact placental angiogenesis throughout gestation.

## Introduction

Fetal growth restriction (FGR), a pregnancy complication where the fetus fails to meet its genetically determined growth potential, is frequently linked to placental dysfunction^1^. FGR affects 5–10% of all pregnancies, but due to limited sensitivity of clinical screening procedures, detecting FGR during pregnancy is challenging, and approximately 30% of FGR remains undetected prior to birth^1^. There are no effective treatments for FGR except close monitoring of the fetus, and thus FGR frequently results in premature delivery^2^. Our inability to accurately predict or effectively treat FGR means that growth restricted fetuses have a perinatal mortality rate eight times higher than normally grown fetuses, due to increased risk of multi-organ dysfunction, and immature metabolic and immune systems^2^. Increased understanding of normal and pathological placental development, and its contribution to FGR, would improve our ability to detect and manage FGR pregnancies.

The placenta is an essential organ for a successful pregnancy since it provides the oxygen and nutrients required for normal fetal growth. It also plays critical roles in adapting the maternal uterine circulation to increase delivery of nutrient-rich maternal blood to its surface, and in absorbing the nutrients from this blood efficiently^3^. To achieve this, the placenta is made up of branching tree-like structures called villous trees^3^. Each branch (or villus) of the tree is encased by an outer bilayer of specialized placental epithelial cells (trophoblasts), that play an important role in the transfer of nutrients and oxygen from the maternal blood into the placenta^3^. In the core of each villus, a network of densely branched blood vessels develops to maximize transfer from maternal to fetal circulations, whilst maintaining a low resistance pathway for fetal blood to be delivered to the site of exchange^3^.

In a normal placenta, *de novo* blood vessel formation (vasculogenesis) takes place from approximately 15 days post-conception^4^. This is followed by angiogenesis: the formation of new blood vessels from pre-existing vessels^4^. Angiogenesis starts around 21 days post-conception and continues until term^4^. Initially, branching angiogenesis is dominant, resulting in the generation of multiple parallel short vessels that increase the vascular exchange area and decrease placental vascular resistance during the first and second trimesters^5^. From the third trimester, non-branching angiogenesis becomes dominant, whereby vessels elongate and coil up to form capillary loops in the terminal villi - the principal sites for feto-maternal exchange^5^. With the right balance between branching and non-branching angiogenesis, normal placentae have a well-branched and multi-looped vascular network^6^. The placental vasculature has been assessed at the micro- and macro-scale using a number of techniques that are comprehensively reviewed by Plitman Mayo^7^. At the macro-scale these include using analysis of vascular corrosion casts^8–10^, computed tomography (CT) using contrast agents in the vasculature^10–12^, and magnetic resonance angiography^13^. At the micro-scale using techniques such as histology^10^, confocal microscopy^14^, micro-CT^12^ and electron microscopy^6^. Studies investigating the placental vasculature in FGR placentae typically show a sparse and elongated vascular network^6,8,10,12^. As a result, placental vascular resistance, which gradually decreases with gestational age in normal pregnancy, remains high in FGR leading to placental insufficiency and adverse fetal outcomes^15^.

Placental vascular development is regulated by a complex network of paracrine factors including vascular endothelial growth factor (VEGF), placental growth factor (PlGF), angiopoietin 1 and 2 (Ang 1&2), fibroblast growth factor (FGF) and platelet derived growth factor (PDGF)^16,17^. Levels of these growth factors are impaired in FGR placentae, and this is thought to contribute to the poor vascular development seen in FGR^17^. Tissue oxygenation also plays an important role in stimulating angiogenesis^18^, and the placenta exists in a physiologically hypoxic environment for the majority of the first trimester, providing an important stimulus for early placental blood vessel development^17,18^. A third key regulator of angiogenesis - mechanical shear stress - is known to play essential roles in angiogenesis in a number of organs including the retina and brain, as well as in pathology (e.g. tumor growth)^13,19^. However, the role of shear stress in regulating placental angiogenesis has been largely overlooked. Consequently, we have no understanding of how shear stress may affect placental vascular malformation in FGR, or conversely how the structural differences we see in the vasculature of FGR placentae may impact shear stress in those vessels.

Direct measurement of the biophysical environment in which placental vasculogenesis and angiogenesis occurs, is impaired by inaccessibility of the placenta to measurement, particularly in early pregnancy^20^. Even if invasive measures were practical, measuring local tissue stress or oxygenation is hampered by the delicate and intricate nature of placental tissue^20^. Computational models allow inference of local tissue properties where direct measurement is not possible^21–23^. Simple models of the fetal circulation in FGR have also been fit to Doppler ultrasound waveforms to elucidate how elevated placental vascular resistance contributes to these waveforms^24,25^. Elevated resistance has been attributed to poor placental vascularization, but as prior models lumped together the whole placental vasculature into one resistance, they could not predict local hemodynamics or shear stress. Recently, our research group has developed an anatomically based computational model of the placental vasculature that includes both macro-level vessels and their interaction with a geometrically idealized capillary structure^21^. This model provides a mechanistic link between macro-scale vascular structure and shear stress in the micro-vasculature, but to date has only been used to investigate normal pregnancy^21^.

In this study, we aimed to analyze the role that shear stress plays in vascular structural malformation in FGR. We hypothesized that vascular structural malformation at the organ level could alter local shear stress, which could subsequently alter the cellular functions driving angiogenesis. To address this, we used a combined computational simulation and *in vitro* approach to demonstrate for the first time that abnormal shear stress in FGR placentae may be the triggering factor of vascular structural abnormalities, and propose that this structure-function defect may interplay during the process of micro-vascular development throughout the gestation where one defect initiates the other.

## Results

### Placental capillary shear stress increases in computational models of FGR

**Figure 1:**
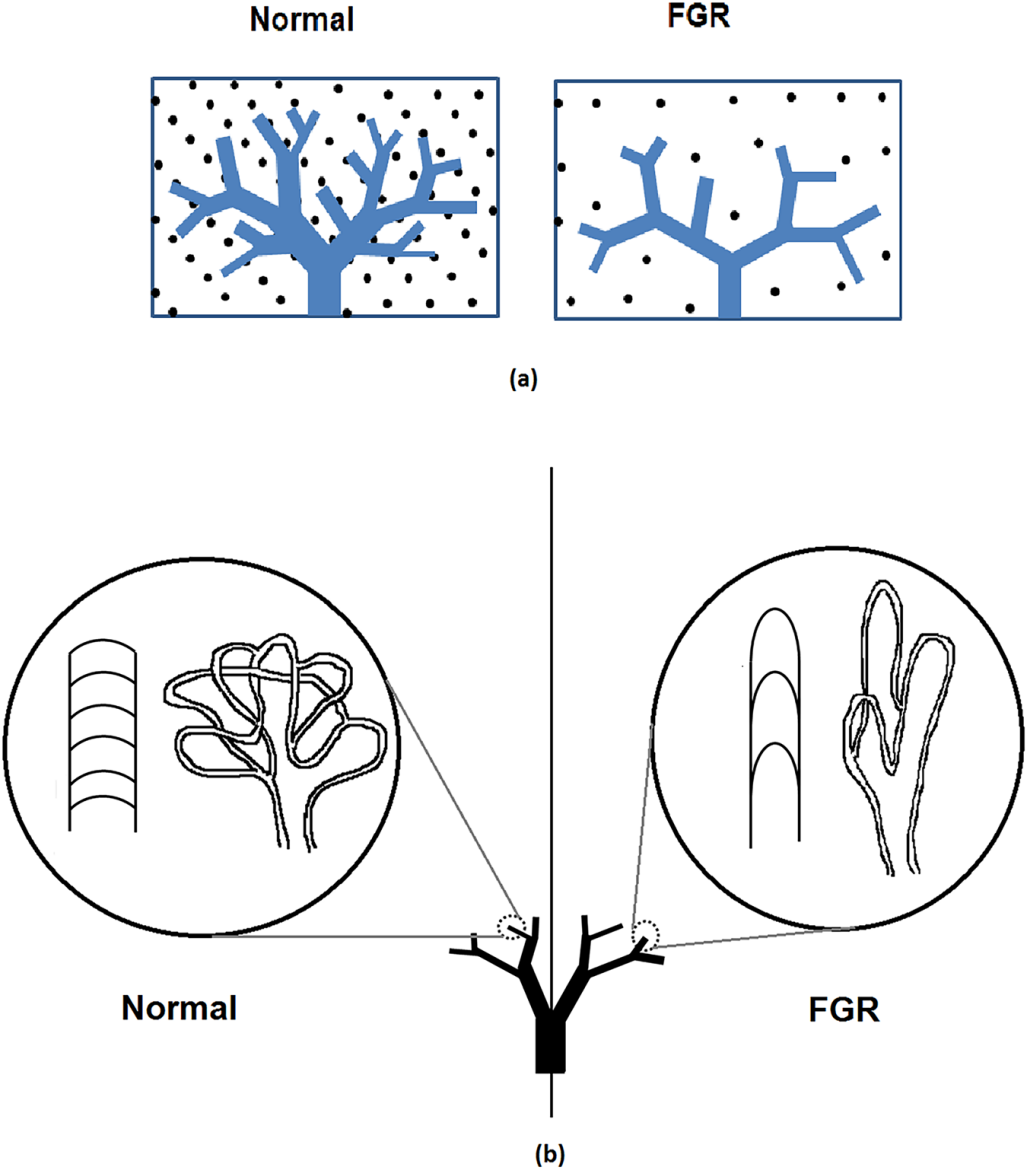
(**a**) A schematic diagram illustrating computational model generation for pre-capillary placental vascular branches. The placental volume is seeded with a fixed density of points, and tree structures are grown toward these points. Sparser seed points result in a sparser vascular tree. (**b**) A schematic diagram of capillary loops that reside distal to intermediate villi and are simplified in the model as a lumped structure. In normal placentae capillary branching is denser than that in FGR placenta.

**Figure 2:**
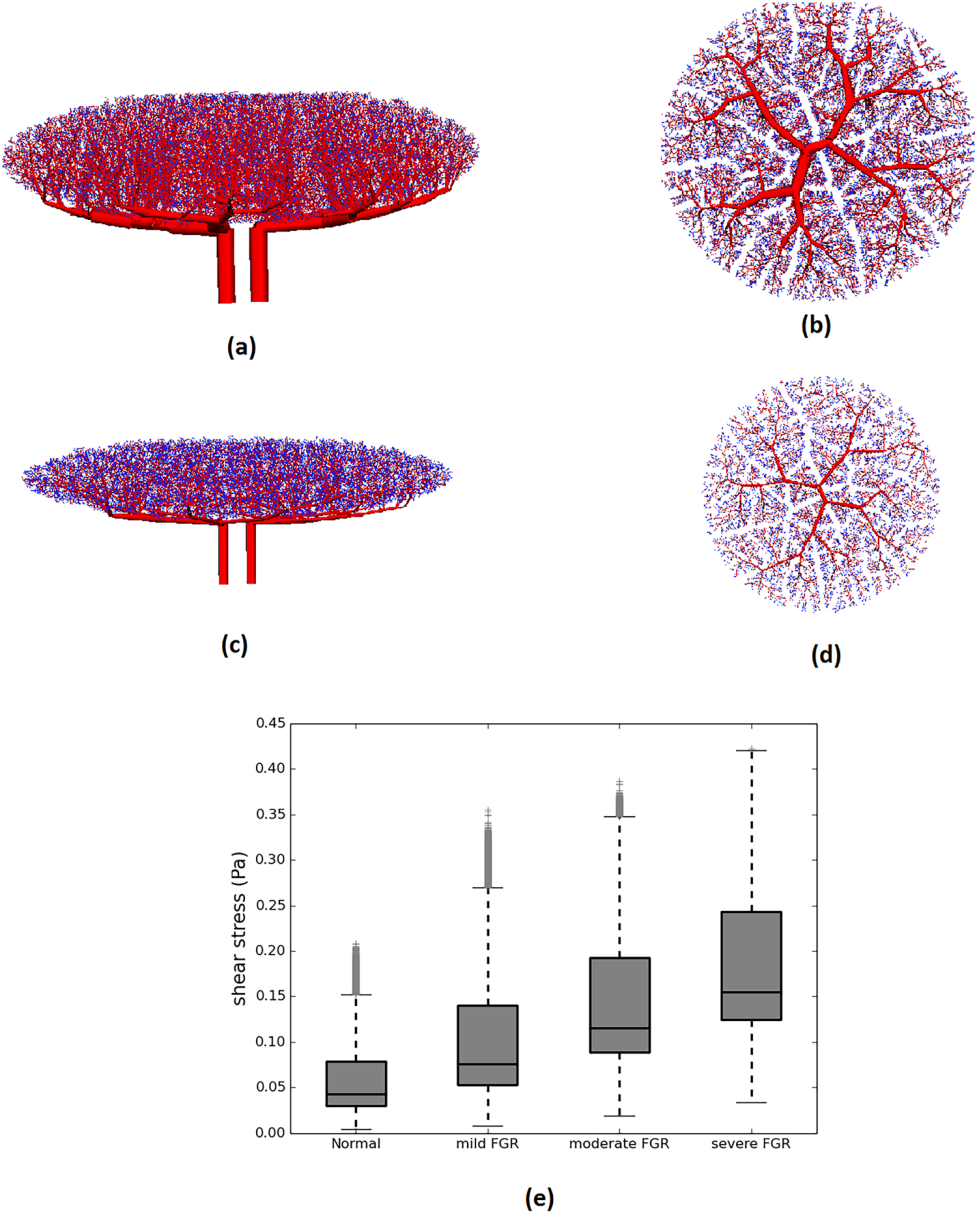
Side (**a**,**c**) and top (**b**,**d**) views of the normal and FGR placental models (pre-capillary level). Normal placentae have an abundant vascular network, whereas FGR placentae are smaller with a sparser vascular network. (**e**) Box and whisker plot showing the median, range and interquartile range of predicted placental capillary shear stress from normal and FGR models. Outlier data points are identified as gray crosses (‘+’).

To understand the relationship between shear stress and blood vessel development in FGR, we simulate the distribution of flow and shear stress in the normal and FGR placental vasculatures (Fig. 1). Our model predicts an increase in volumetric blood flow per capillary in FGR placentae: 0.22 *µ*l/min (mild), 0.31 *µ*l/min (moderate) and 0.40 *µ*l/min (severe) compared to 0.13 *µ*l/min in normal placentae. Consequently, mean capillary shear stress is elevated in our models of FGR placentae: 0.10 Pa (mild), 0.14 Pa (moderate) and 0.19 Pa (severe) in comparison to 0.06 Pa for normal placentae (Fig. 2e).

### Endothelial cell migration is more persistent under FGR shear stress conditions

Angiogenesis involves a balance in endothelial cell migration speed, persistence and proliferation^16^. During angiogenesis, endothelial cells migrate towards stimuli for both vessel sprouting and elongation, therefore, adequate *speed* of cell migration is essential for vascular development^26^. *Persistency* determines the efficiency of angiogenesis; highly persistent cells tend to lead to vessel elongation rather than branching^27,28^. Endothelial cells also need to *proliferate* sufficiently to support new vascular development^16^. Therefore, to understand how the computationally predicted capillary shear stress affects placental angiogenesis, endothelial cells were exposed to these shear stresses and their speed, persistence, and proliferation were analyzed.

**Figure 3:**
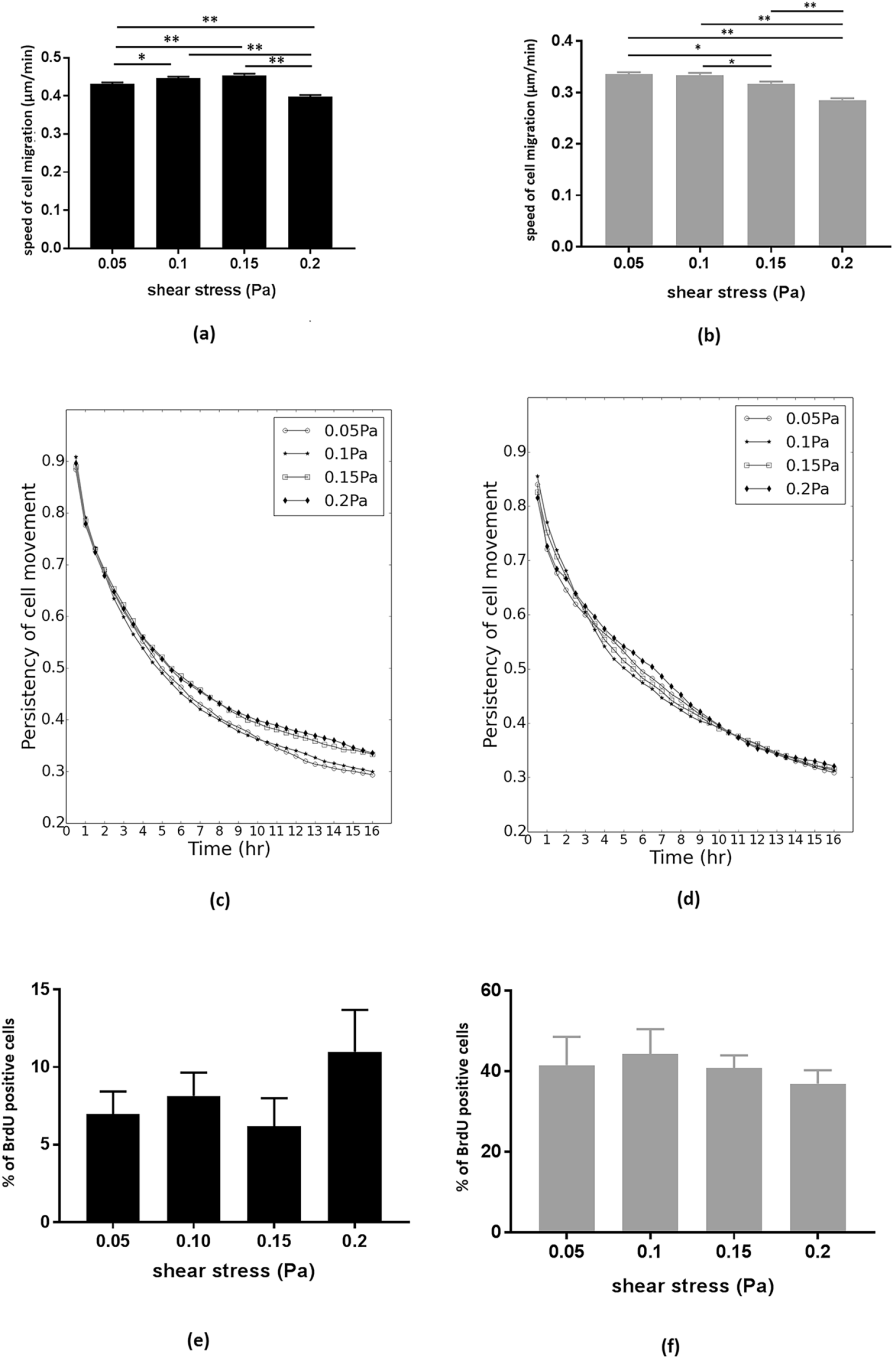
Bar graphs showing the mean migration speed of HUVECs (**a**) and HMEC-1 (**b**) under shear stress of normal (0.05 Pa) and FGR conditions (0.1, 0.15 and 0.2 Pa). Error bars represent the standard error (* and ** show statistical significance, *p = 0.01, **p < 0.0001). Line graphs showing the persistency of HUVECs (no significant differences) (**c**) and HMEC-1 (0.05 Pa and 0.1 Pa are significantly less persistent than 0.15 and 0.2 Pa, *p* < 0.001) (**d**) under shear stress. Bar graphs showing the proportion of HUVECs (**e**) and HMEC-1 (**f**) that incorporated BrdU over a 4 hr period.

In HUVECs, the speed of endothelial cell migration was significantly higher when exposed to shear stress predicted in capillaries of mild (0.1 Pa, p = 0.01) and moderate (0.15 Pa, p < 0.0001) FGR compared to that predicted in capillaries of normal placentae (0.05 Pa). However, when exposed to the shear stress predicted in capillaries of severe FGR placentae (0.2 Pa), HUVEC migration was significantly slower than normal (0.05 Pa), mild FGR (0.1 Pa) and moderate FGR (0.15 Pa) (all p < 0.0001) (Fig. 3a). In HMEC-1, the speed of cell migration decreased consistently and significantly when exposed to shear stress predicted in capillaries of moderate (0.15 Pa) and severe (0.2 Pa) FGR compared to that of normal (0.05 Pa) and mild FGR (0.1 Pa) (P <  = 0.01) (Fig. 3b).

HMEC-1 were significantly more persistent when exposed to shear stress predicted in capillaries of moderate (0.15 Pa) and severe (0.2 Pa) FGR placentae than lower shear stress of normal (0.05 Pa) and mild FGR placentae (0.1 Pa) with p < 0.001 in each case (Fig. 3d). In contrast, macrovascular HUVECs exhibited no significant difference in persistency between any of the shear stress conditions employed (p > 0.99), shown in Fig. 3c. There was no significant difference in the proportion of cells that incorporated BrDU (indicating cell proliferation) between any of the shear stress conditions employed for either HUVECs (n > 820 cells quantified in each of the 4 shear stress conditions, totaling 43830 cells) or HMEC-1s (n > 900 cells quantified in each of the 4 shear stress conditions, totaling 29538 cells) (p > 0.99), shown in Fig. 3e,f.

## Discussion

In this study we probed the role of placental vascular architecture on placental hemodynamics and angiogenesis via computational modeling, *in vitro* and anatomical techniques. By combining whole organ analysis of hemodynamics and mechanical stress, cell level behaviors under these stresses, and anatomical structure, we are able to integrate macro- and micro-scale circulatory function in placental development. Our computational model shows that both the macro- and micro-scale vasculature contribute to placental vascular shear stress, and our *in vitro* data shows that the endothelial cell functions contributing to angiogenesis are significantly impaired when endothelial cells are exposed to abnormally high shear stress representative of FGR placentae. Finally, we have demonstrated that these cell-level behaviors are consistent with changes in placental vascular anatomy in FGR, showing that that placental vascular shear stress is likely an important driver of vascular malformation in FGR placentae.

During pregnancy, placental vascular abnormalities are typically detected by clinical screening only when there are extensive structural abnormalities in the macro-vasculature^29^. Micro-vascular abnormalities are often undetectable during pregnancy; hence, we have no clear knowledge of the functional effects of micro-structural abnormalities on placental hemodynamics and angiogenesis. By simulating abnormalities in macro-vascular structures alone (mild FGR case) and then progressively increasing the level of pathology in the micro-vasculature (moderate and severe FGR cases) we are able to separate the impact of these structural abnormalities. Our model predicts that the impact of pre-capillary vascular structural alterations that are representative of FGR is to increase total placental vascular resistance by approximately 5 times (1.02 Pa/mm^3^/sec in normal and 5.49 Pa/mm^3^/sec in mild FGR). The model predicts that this would lead to an elevation of capillary level shear stress to almost double normal values (0.06 Pa in normal and 0.10 Pa in mild FGR). However, endothelial cell function does not appear to be altered under these conditions. When structural abnormalities occur in both the macro- and micro-vasculature (i.e. moderate and severe FGR scenarios), and placental capillary loop formation is sparse, our model predicts further increases in placental vascular resistance and shear stress. With increasing capillary sparsity our model predicted increases in micro-vascular shear stress of up to 0.19 Pa in our severe FGR model, which is approximately 3 times greater than predicted in normal placentae. These increased shear stresses in moderate and severe FGR conditions are sufficient to impact on endothelial cell function. This finding suggests that vascular structural defects localized to the micro-vessels (for instance reduced density and dimension of placental capillaries) are critical factors determining the placental vascular shear stress and endothelial cell function. As the pathology of FGR begins early in gestation as the vascular trees are forming, it is likely that these structure-function maladaptations may be evident from early in gestation and continue throughout pregnancy.

Angiogenesis occurs in three principal steps: (1) degradation of the vascular basement membrane when exposed to stimuli (for example, VEGF, mechanical stress) (2) endothelial cell proliferation and migration towards stimuli to form a cord like structure (3) lumenization of the cord to form a new vessel^16^. Therefore, endothelial cell migration and proliferation are fundamental steps in initiating new vascular branches. The effect of shear stress on endothelial cell proliferation and migration has been examined previously using macro-vascular shear stress (0.5–5 Pa)^30,31^. However, no study has employed micro-vascular shear stress (0.05–0.2 Pa), or shear reflective of the placental vasculature. For the first time, our computational model made it possible to predict the *in vivo* micro-vascular shear stress of normal and FGR placentae and examine endothelial cell function *in vitro* under these conditions.

Cell migration is essential for angiogenesis as it drives vessel development towards stimuli that promote vascular network formation^32^. Indeed, cell migration rate is thought to be the primary determinant of the rate of vascular network growth^32^, and angiogenesis is significantly impaired when the speed of cell migration is reduced^26,33^. Therefore, slower endothelial cell migration, as seen in shear stresses predicted in severe FGR placentae in this work, would interfere with normal angiogenesis, leading to a reduced placental vascular network formation. Another important factor for placental angiogenesis is a balance between random and persistent endothelial movement, since random movement is required for branching angiogenesis, while persistent movement drives vessel elongation^27,28,32,34^. Indeed, mutations in genes controlling either random (CXCR7) or persistent (Borg5) migration of endothelial cells impede angiogenesis^27,34^. Here, the persistency of micro-vascular endothelial cells significantly increases when exposed to abnormally high shear stress reflective of moderate and severe FGR cases, which could lead to the formation of a more elongated placental capillaries with fewer branches. We see in conditions of elevated micro-vascular shear stress that micro-vascular endothelial cells in particular migrate more slowly, and in a more persistent manner. We propose that this leads to less branching angiogenesis and sparser, longer capillary network, as detected in anatomical studies^6,18^. This phenomenon could further aggravate placental capillary deformities, further elevating shear stress, and further impacting capillary branching. This whole process may continue throughout pregnancy, reinforcing a negative cycle of impaired vascularization.

Although shear stress has been previously reported to stimulate endothelial cell proliferation^31,35^, these studies employed shear stress at levels typical of the macro-vasculature. Under shear stresses predicted in the placental microvasculature we did not see a significant effect on cell proliferation in either HUVEC or HMEC-1. However, as shear stress increased within this range we saw a significant decrease in cell migration speed in both HUVEC and HMEC-1s, and increase in persistence in HMEC-1s only. Taken together this suggests that elevated shear stress contributes to angiogenesis at the microvascular level primarily by influencing cell migratory behavior rather than cell proliferation. HUVECs (macro-vascular) are typically exposed to higher rates of blood flow, and higher shear stress than HMEC-1 (micro-vascular); therefore, HUVECs may be less sensitive to a low-level capillary shear stress typical of the micro-vasculature, which may relate to their diminished response to low shear in this work.

Analyses of placental vascular structure have shown smaller placental chorionic plate surface area and a higher vascular length density in FGR placentae compared with normal placentae^10,18^. Our simulations indicate significantly elevated vascular resistance in FGR structures (1.02 Pa/mm^3^/sec, 5.49 Pa/mm^3^/sec, 6.94 Pa/mm^3^/sec, 8.30 Pa/mm^3^/sec in normal, mild, moderate and severe FGR respectively). As from the definition of resistance in a tubular structure such as a blood vessel (Equation 1), the smaller diameter and longer vascular structures seen in FGR placentae contribute to elevated placental vascular resistance which leads to abnormal Doppler flow patterns (absent or reverse end-diastolic placental blood flow (EDF)) in FGR^36^. However, our model allows estimation of the extent of increases placental vascular resistance that may be seen in placentae with vascular structures typical of FGR, allowing quantification of the impact of observed anatomy on function. Models of oxygen exchange, linking villous branching to oxygenation also suggest that longer branching structures are suboptimal^37^. Thus, the vascular structures typical of FGR are likely detrimental to both the resistance of the feto-placental vasculature and its exchange function. As our model shows that significant differences in normal and FGR vascular resistance can be attributed to the macro-vasculature, analysis of the chorionic structure may be an important tool in distinguishing between normal and abnormally branching placentae. However, the complex structure of micro-vasculature, which can typically only be imaged *ex vivo* post-delivery^12,14,38^, is predicted to be a major contributor to the shear response of placental vascular endothelial cells, which could impact on ongoing placental vascular development.

### Limitations

The parameterization of micro-vascular structure in models is made based on data acquired from the literature, and not an analysis of macro-vascular and micro-vascular structure in the same placenta. To do this would require multi-scale imaging, designed to capture the structure of the feto-placental vasculature from umbilical artery to capillary. Thus, the model structures do not necessarily reflect an individual placenta diagnosed with FGR. The grading of severity of vascular structural abnormalities in this study therefore does not reflect a clinically determined severity, and instead aims to cover the range of possible scenarios based on our own data and data reported by others. The real FGR placenta is likely somewhere on this spectrum of pathology and perturbing a computational model in this way allows assessment of which components of the vasculature contribute to function in a manner that can be readily quantified.

The model of placental haemodynamics makes a number of assumptions, discussed in detail previously^21^. Specifically, blood flow is assumed to be governed by equations that represent incompressible, Newtonian, laminar flows with negligible impact of disturbances at bifurcations, and due a paucity of data regarding placental vascular compliance the effect of elasticity of vascular network was ignored. In addition, placental capillary structure was simplified using a lumped model, which includes a symmetrically branching network of arterioles, venules and capillaries, but does not include the geometric accuracy of computational studies focusing on micro-scale placental structures (e.g.^22,38^). These geometrical assumptions are needed to simulate blood flow through the whole vasculature, including macro- and micro-scale structure, but more detailed measurements across scales and comparison of simplified models to more anatomically detailed models, particularly at the micro-scale could improve the accuracy of the model in the future.

## Conclusions

In FGR, organ level structural abnormalities of placental vessels are often apparent at delivery. These abnormalities impact on the distribution of blood flow and mechanical stress in the placental vascular system. However, these functional consequences of vascular pathology are difficult to investigate *in vivo*. We have overcome this by using a computational model to predict *in vivo* function, and shown that mechanical shear stress is likely elevated in the placental micro-vasculature of FGR pregnancies. As a consequence, endothelial cell function is negatively affected and angiogenesis is disrupted, potentially compromising the subsequent micro-vascular network formation. Our data suggests that this process may occur throughout pregnancy where the vascular structural and functional aberrations act as cause and consequence of each other.

## Methods

### Simulation of placental hemodynamics

To date, there is no invasive or imaging technology that can accurately measure the *in vivo* placental capillary shear stress. Here we apply computational modeling to predict *in vivo* capillary shear stress in both normal and FGR placentae. We employ a previously published computational model of the placental vasculature^21^. Details of morphometric model generation are provided as Supplementary Information, with key features of the model outlined here. All models were constructed and implemented using CMISS and CMGUI (www.cmiss.org), open-source software designed specifically for modelling biological systems.

Each blood vessel to the level of intermediate villous vessels was explicitly represented as a one-dimensional element, generated to fill a spheroidal placental volume and defined by two nodes (a start point and end point), and the radius of the vessel in question^21^. These vessels are generated over the chorionic surface using an area-filling branching algorithm, and into the placental volume using an analogous volume-filling branching algorithm. The algorithms are parameterized to match placental vascular branching parameters, as measured in morphometric studies^6,8,10,39–41^. The volume-filling algorithm is modified to FGR placental structure in this study. The volume-filling branching algorithm fills the placental volume with ‘seed points’ distributed to represent vascular density, splits the seed points by geometrical features of the existing branching vasculature, and iteratively repeats the process until either no seed points remain, or the generated vessels are below a threshold size (Fig. 1a). The parameters that drive generation of these anatomically based vascular branching geometries are placental volume (*υ*), placenta thickness (*τ*), the number of villous trees (*n*_*v*_) and a metric of vascular density, which determines the number of intermediate villous vessels, *n*_*i*_ in the placenta. Feto-placental veins are generated to match this arterial tree, and the feto-placental vasculature distal to the intermediate villous vessels are incorporated by taking a ‘lumped’ approach. In this lumped model, each intermediate villous vessel supplies three symmetric generations of terminal loops^41^ which contain *n*_*c*_ capillaries that reside in what are termed terminal villi, the site for gas exchange (Fig. 1b).

Studies of placental morphology in FGR suggest a reduction of placental volume by approximately 35% (range 20–45%)^8,39^, a reduction in placental thickness of 25% (range 10–40%)^42^ and a reduction in the number of cotyledons by approximately 45% (range 25–65%)^9^. In addition, vascular density is reduced by 10% (range 5–10%)^10^ and arterial and venous diameters across the placenta are reduced by 20% (range 15–20%)^8^. A critical contributor to FGR is likely impaired micro-vascular development and capillary looping is reduced in FGR (as illustrated in Fig. 1b)^6^. Based on these population-based data, we therefore simulate four scenarios (1) a normal placenta (parameterized as in^21^), (2) a mild FGR placenta with normal micro-vasculature but a typical FGR macro-vasculature, (3) a moderate FGR placenta with reduced micro-vascular branching (*n*_*c*_ decreased by one-third compared with normal) with typical FGR macro-vasculature (4) a severe FGR placenta with further reduced micro-vascular branching (*n*_*c*_ decreased by one-half compared with normal) and typical FGR macro-vasculature. The classification of mild, moderate and severe FGR is based on increasing severity of vascular impairment parameterized in our models, rather than a clinical classification, as vascularization (particularly micro-scale features) is typically not measured clinically. This parameterization thus reflects our primary goal to understand the impact of vascular defects at different levels of placental blood vessels on capillary shear stress, rather than an attempt to reflect clinical severity. Parameter values that have been altered from the normal pregnancy case reported by Clark *et al*.^21^ to reflect FGR pregnancies are summarized in Table 1 and a complete description of the model and a list of model parameters is given as Supplementary Information. Examples of generated models are shown in Fig. 2(a–d).

**Table 1:** Parameters used to generate normal and pathological placental hemodynamic models.

Parameter	Normal	Mild FGR	Moderate FGR	Severe FGR	Reference
Placental volume, *υ*	428 cm^3^	↓35%	↓35%	35%	^8, 21, 39^
Placental thickness, *τ*	20 mm	↓ 25%	↓25%	↓25%	^21, 42, 45^
Number of villous trees, *n*_*v*_	70	↓45%	↓45%	↓45%	^9, 21^
Vascular seed point density	75/cm^3^	↓10%	↓10%	↓10%	^10^
Number of capillaries per terminal villous, *n*_*c*_	6	As normal	↓33%	↓50%	^6, 21^
Umbilical artery radius	2 mm	↓20%	↓20%	↓20%	^8, 21^
Umbilical vein radius	4 mm	↓20%	↓20%	↓20%	^8, 21^

To simulate the impact of feto-placental vascular structure, total placental volumetric blood flow was assumed constant for both normal and FGR scenarios and fixed at 250 ml/min, with umbilical vein blood pressure fixed in all simulations at 20 mmHg (2660 Pa)^21^. By assuming that blood flow is Newtonian, steady, laminar, and that disturbances to flow at vessel bifurcations are negligible, placental vascular resistance is calculated using an electrical analogue approach^21^, with Poiseuille’s law used to determine resistance, *R*, in each vessel

1 $$R=\frac{8\mu L}{\pi {r}^{4}},$$

where *μ* is the viscosity of blood (3.36×10^*−*3 ^Pa.s)^43^, *L* is the length of the blood vessel and *r* is its radius. The resistance of the entire system is calculated by summing resistances through the network in series and parallel, assuming mass conservation at bifurcations. This allows total resistance, and the distribution of pressure and volumetric flows through each branch in the system to be calculated. By defining the flow boundary condition at inlet and outlet elements, the volumetric flow distribution through the geometry was solved. Since a linear relationship between pressure and vessel radius was implemented in our model, the simulated flow is laminar in nature. Shear stress in each blood vessel is then calculated using

2 $${\rm{shear}}\,{\rm{stress}}=\frac{4\mu Q}{\pi {r}^{2}l},$$

where *Q* is volumetric blood flow through that vessel. As feto-maternal exchange primarily occurs at the micro-vasculature, our main interest in this study was developmental process of placental capillaries, therefore, we focus on predicting micro-vascular shear stress in the capillary network.

### Endothelial cell shear stress assays

Once micro-vascular shear stress was predicted by computational modeling, both micro-vascular (human micro-vascular endothelial cell line - HMEC-1) and macro-vascular (primary human umbilical vein endothelial cells - HUVECs) endothelial cells were exposed to the range of predicted micro-vascular shear stress using a microfluidic system as previously described^44^.

Briefly, umbilical cords of normal placentae were collected from Delivery Suite at Auckland City Hospital following informed consent with ethical approval from the Northern X Ethics Committee (NTX/12/06/057/AM06). Primary HUVECs were isolated from these cords as described in^44^. HMEC-1 cell lines were purchased from ATCC. HUVECs (n=3 biological replicates, with 4 technical replicates in each shear stress condition) or HMEC-1 (n=8 technical replicates for each shear stress condition) were suspended at 10 million cells/mL, and 20 *µ*L of this suspension was seeded into fibronectin coated channels of a Bioflux200 microfluidic shear stress culture system (Fluxion, USA) by applying a pulse of shear stress at 0.3 Pa for 10 seconds, which ‘pushed’ a portion of cells from this droplet into the microfluidic channel at a sufficient density to create a monolayer of endothelial cells. It is important to note that the number of cells seeded into the inlet well does not directly equate to the number of cells in the channel, but this protocol was previously optimised to ensure the correct cell density in the channel to create a confluent monolayer. 200 *µ*L of 1:1000 CellMask^*TM*^ Orange plasma membrane stain (Life Technologies, New Zealand) diluted in culture medium [either a 50:50 mixture of RPMI1640 (ThermoFisher Scientific, New Zealand) and M199 (ThermoFisher Scientific, New Zealand) containing 20% FBS (fetal bovine serum), 100U/ml penicillin-streptomycin, 2.9 mg/ml L-Glutamine, 20 *µ*g/ml endothelial cell growth supplement and 1 *µ*g/ml heparin for HUVECs, or MCDB131 (Life Technologies, New Zealand) containing 10% FBS, 100U/ml penicillin-streptomycin, 2.9 mg/ml L-Glutamine for HMEC-1] was infused into each channel at a rate of 0.1 Pa (1dyne/cm^2^) for 2 min. The cells were then incubated at 37 °C for 10 min. Channels were washed by infusing phosphate buffer saline (PBS) into each channel for 10 min at a shear of 0.1 Pa. 3 ml of pre-gassed media was added into each inlet well and the cultures were exposed to 0.05, 0.1, 0.15 or 0.2 Pa of shear stress representing computationally predicted values for normal, mild, moderate and severe FGR respectively. Shear stress was applied to cultures for 16 hours at 37 °C with phase contrast and fluorescent images captured automatically every 15 minutes using a Nikon TE-2000 inverted fluorescence microscope, housed within a Solent microscope incubation chamber to ensure a consistent 37 °C environment.

#### Endothelial cell migration

To quantify endothelial cell migration, HUVECs from 60 image sequences (approximately 4800 cells in total), and HMEC-1 from 60 image sequences (approximately 5500 cells in total) were analyzed by manual cell tracking by a single observer. To do this, the location of each cell center was tracked from frame-to-frame using the Fiji (https://fiji.sc/) ‘manual tracking plugin’ (https://imagej.nih.gov/ij/plugins/track/track.html). A Matlab (version R2013a, The Mathworks Inc) algorithm was developed to calculate the speed and distance traveled by each cell from frame to frame. Persistency in cell movement was calculated by dividing the straight-line distance between start and end points by the actual distance traveled by the cells for each frame. Values close to one indicate that the total distance travelled over time is similar to the difference between start and end point, and so cells move persistently in the same direction.

#### Endothelial cell proliferation

The proliferation rate of endothelial cells following exposure to shear stress was determined by bromodeoxyuridine (BrdU) incorporation. To do this, following 16hrs of shear stress, 200 *µ*l of 1:10 BrdU (Becton Dickinson, Australia/New Zealand) was infused into the Bioflux channel for 15 min at 0.1 Pa and the culture was incubated for 4 hr at 37 °C. Endothelial cells were fixed by introducing 4% paraformaldehyde (PFA) for 20 min at 0.1 Pa. Channels were washed with 0.1% Triton X for 10 min at 0.1 Pa, then 10%FBS (fetal bovine serum) in PBS for 30 min at 0.1 Pa. 200 *µ*l of DNase (300 *µ*g/ml) was infused into the channel for 15 min at 0.1 Pa, and incubated for 1 hr at 37 °C. 200 *µ*l of 1:10 anti-BrdU antibody (Becton Dickinson, Australia/New Zealand) was added into the inlet well and infused through the channel for 1.5 hr at 0.1 Pa. The channels were washed with PBS and nuclei were counter-stained with 2 *µ*g/ml Hoechst 33342. Cells were imaged using a Nikon TE-2000 fluorescent microscope and cell proliferation was quantified by digital image analysis of the number of BrdU positive cells using ImageJ. Data were analyzed by one-way ANOVA using GraphPad Prism 7. Data are reported as means and standard errors, and p-values < 0.05 considered as statistically significant.

## Supplementary information

Computational model of feto-placental hemodynamics

## Data Availability

The datasets in the current study are available from the corresponding author on reasonable request.
