# Supplementary material for: Differences in placental capillary shear stress in fetal growth restriction may affect endothelial cell function and vascular network formation
Source: Sci Rep. 2019 Jul 8;9:9876. doi: 10.1038/s41598-019-46151-6 (PMC6614400; doi:10.1038/s41598-019-46151-6)
Supplement: Supplementary file 1 — Computational model of feto-placental hemodynamics [file 41598_2019_46151_MOESM1_ESM.pdf]

Differences in placental capillary shear stress in fetal growth restriction may affect endothelial cell function and vascular network formation.

Win M Tun[1], Choon Hwai Yap[2], Shier Nee Saw[2], Joanna L James[3], and Alys R Clark[1,*]

[1]Auckland Bioengineering Institute, University of Auckland, New Zealand
[2]Department of Biomedical Engineering, National University of of Singapore, Singapore
[3]Department of Obstetrics and Gynaecology, Faculty of Medical and Health Sciences, University of Auckland, New Zealand
[*]alys.clark@auckland.ac.nz

1 Computational model of feto-placental hemodynamics

The computational model of feto-placental hemodynamics employed in this study was first published by Clark et al.[1], and the model is constructed as described in that study. The key steps in generating this model are (1) defining the shape and volume of the placenta, (2) using surface and volume filling branching algorithm to generate a branching vascular model within the placental volume, (3) defining a lumped parameter model for the placental micro-vasculature, (4) simulating blood flow within this branching network of blood vessels, and (5) calculating micro-vascular shear stress based on blood vessel size and regional blood flow. All models were constructed and implemented using CMISS and CMGUI (www.cmiss.org), open-source software designed specifically for modelling biological systems.

1.1 Defining the shape and volume of the placenta

In this study, as in the study of Clark et al.[1], the shape of the placenta is assumed to be adequately represented as an spheroidal shape. The dimensions of the placenta can then be determined by its volume (υ), and thickness (τ) using

$$\upsilon = \frac{4}{3}\pi r^2 \tau, \tag{1}$$

where r is the radius of the placenta (a chorionic plate equivalent radius). The placental volume is represented by a finite element surface mesh[1,2].

1.2 Using surface and volume filling branching algorithm to generate a branching vascular model within the placental volume

The chorionic vessels: The vascular branches that cover the chorionic plate are generated to lie on one surface of the finite element mesh representing the placental volume. Where data from imaging are not directly used, a chorionic vasculature is generated by generating uniformly distributed points that cover this surface, and using a Monte Carlo method defined by Wang et al.[3]. The number of seed points is equivalent to the number of villous trees, n_v.The umbilical arteries are defined to meet the chorionic plate at its centre, and to be a distance of 10 mm apart. Then, the following 3 steps are repeated until there are no seed points remaining: 1) for each current terminal branch seed points are slot by the line between that branch and the centre of mass of the seed points, 2) The centres of mass of the two new seed point regions are calculated, 3) two new branches grown a fixed distance from the end of the current terminal branch toward the centres of mass of the two seed point regions. If data showing chorionic vessel distribution is available, these vessels are mapped onto the 2D plane defining the chorionic surface of the placenta.

The villous trees: A similar method is used to define the geometry of the villous trees. Except, instead of covering a surface, blood vessel elements are defined to fill the volume of the placenta, using a method described by Tawhai et al.[4]. The volume-filling branching algorithm fills the placental volume with 'seed points' distributed to represent vascular density, splits the seed points by geometrical features of the existing branching vasculature, and iteratively repeats the process until either no seed points remain, or the generated vessels are below a threshold size (0.1 mm).

Terminal convolutes: The model of Clark et al.[1] represents all blood vessels distal to a mature intermediate villous in a lumped-parameter model. Although each vessel is represented explicitly in resistance calculations, the structure of the terminal convolutes is simplified to be represented as a symmetrically branching tree with n_c capillary connections at each level of the tree. There are assumed to be three symmetric generations of immature intermediate villi within this lumped parameter structure, each with n_c capillaries connecting arterioles to venules at this level.

1.3 Defining vessel dimensions

To define the calibre of each vessel element in the vascular network we define the radius of the largest arteries (the umbilical arteries) and vein (the umbilical vein) and then define the radii of the arteries and veins, as two separate groups, by using Strahler branching rules. To do this the smallest vessels in the branching component of the model (mature intermediate villi) are defined as order 1 vessels, and then moving recursively up the tree at each bifurcation the vessel upstream of that bifurcation is prescribed either 1) the maximum order of its child vessels (if the orders of the child vessels are different) or 2) the order of its child vessels plus one (if the order of its children are equal). Each branch in the network is then defined a diameter using

$$\log D(x) = (x-N)\log R_{dS} + \log D_N, \tag{2}$$

where $D(x)$ is diameter of all vessels of order x, N is the highest order of any vessel in the network, R_{dS} is a Strahler diameter ratio, and D_N is the diameter of vessel at order N (here the umbilical artery/vein).

1.4 Simulating blood flow distribution

The placental vasculature is represented as a network of cylindrical tubes, and blood flow within them is assumed to be steady state, in compressible, laminar, and with negligible disturbances at bifurcations. This allows simplification of the governing Navier-Stokes equations for fluid flow to a Poiseuille equation. The resistance, R, of each blood vessel is defined as

$$R = \frac{8\mu L}{\pi r^4}, \tag{3}$$

where L is the length of the vessel, $\mu = 3.36 \times 10^{-3}$ Pa.s is the viscosity of blood, and r is the radius of the vessel. The pressure drop, ΔP, in each vessel is related to the volumetric flow of blood, Q in that vessel by

$$\Delta P = QR. \tag{4}$$

One can then consider the network of blood vessels in the placenta as an equivalent network of resistors, and resistance is summed in series and parallel to determine the total resistance of the system. Pressure and flow distributions through the system are also calculated assuming conservation of flow at bifurcations (flow in equals flow out), and continuity of pressure at bifurcations. These calculations are performed in CMISS (www.cmiss.org).

Table 1. Geometric parameters defining normal model of the placental vasculature. Data sources are outlined in Clark et al.[1].

Parameter	Description	Value
υ	Placental volume	428 cm^3
τ	Placental thickness	2.0 cm
n_v	Number of villous trees	70
	Vascular seed point density	75 /cm^3
n_c	number of capillaries per intermediate villous	6
r_{UA}	Radius of umbilical artery	2 mm
r_{UV}	Radius of umbilical vein	4 mm
r_{iv}	Radius of mature intermediate villous	0.015 mm
l_{iv}	Length of mature intermediate villousa	1.5 mm
r_c	Radius of terminal capillary	0.0072 mm
l_c	Length of terminal capillary	3 mm
	Radius of placental veins	twice arterial diameter
R_{dS}	Strahler diameter ratio	1.53

References

1. Clark, A. R., Lin, M., Tawhai, M., Saghian, R. & James, J. L. Multiscale modelling of the feto-placental vasculature. *Interface Focus.* **5**, 20140078 (2015).
2. Fernandez, J., Mithraratne, P., Thrupp, S., Tawhai, M. & Hunter, P. Anatomically based geometric modelling of the musculo-skeletal system and other organs. *Biomech. modeling mechanobiology* **2**, 139–155 (2004).
3. Wang, C., Bassingthwaighte, J. & Weissman, L. Bifurcating distributive system using monte carlo method. *Math. computer modelling* **16**, 91–98 (1992).
4. Tawhai, M. H., Pullan, A. & Hunter, P. Generation of an anatomically based three-dimensional model of the conducting airways. *Annals biomedical engineering* **28**, 793–802 (2000).
